# Nuclear mechanotransduction: tools for mechanical perturbation and chromatin characterization

**DOI:** 10.1080/19491034.2026.2690847

**Published:** 2026-07-06

**Authors:** Jennifer Soto, Nolan Origer, Yifan Wu, Braulio Cardenas Benitez, Ramzi Massad, Donna Rahgoshay, Abraham Lee, Timothy Downing, Song Li

**Affiliations:** a Department of Bioengineering, University of California Los Angeles, Los Angeles, CA, USA; b Department of Biomedical Engineering, University of California Irvine, Irvine, CA, USA; c NSF-Simons Center for Multiscale Cell Fate Research, University of California Irvine, Irvine, CA, USA; d Edwards Lifesciences Cardiovascular Innovation and Research Center, University of California Irvine, Irvine, CA, USA; e Sue & Bill Gross Stem Cell Research Center, University of California Irvine, Irvine, CA, USA; f Department of Chemistry and Biochemistry, University of California Los Angeles, Los Angeles, CA, USA; g Department of Microbiology and Molecular Genetics, University of California Irvine, Irvine, CA, USA; h Center for Complex Biological Systems, University of California Irvine, Irvine, CA, USA; i Department of Medicine, University of California Los Angeles, Los Angeles, CA, USA; j Eli and Edythe Broad Center of Regenerative Medicine and Stem Cell Research, University of California Los Angeles, Los Angeles, CA, USA; k Jonsson Comprehensive Cancer Center, David Geffen School of Medicine, University of California Los Angeles, Los Angeles, CA, USA

**Keywords:** Mechanobiology, nuclear mechanics, epigenetic regulation, biomaterials, microfluidics, single cell imaging

## Abstract

Mechanical cues, ranging from matrix mechanical properties to dynamic mechanical loading, can be transmitted via structural proteins and signaling molecules to the nucleus to reorganize nuclear architecture and modulate chromatin accessibility. This mechanical regulation plays an important role in tissue regeneration and disease development. To gain deeper insights into the mechanical regulation of chromatin organization, it is essential to develop technologies that can apply mechanical inputs and characterize the resulting changes in nuclear structure and chromatin organization. Here, we review multidisciplinary technologies and tools that enable mechanical perturbation of the nucleus and the characterization of nuclear and chromatin responses. We highlight how perturbations such as matrix topography, confinement, stiffness, viscoelasticity, and dynamic loading can be used to apply mechanical cues to cells. We also discuss how imaging-based techniques, sequencing platforms, and computational approaches can be integrated to characterize nuclear architecture and chromatin organization in response to these mechanical stimuli.

## Introduction

Within tissue microenvironments, cells encounter diverse mechanical cues originating from the fluid phase, including shear stress and pressure, and from the extracellular matrix (ECM), including substrate stiffness, viscoelasticity, topographical features, physical confinement, and dynamic loading (Table 1) [1–4]. Advances in mechanobiology and cell engineering have revealed that such physical inputs regulate nuclear mechanics, reorganize chromatin, reshape epigenetic landscapes, and modulate gene expression and cell phenotype [5–7]. These mechanically induced changes can in turn regulate morphogenesis, tissue remodeling, and disease development [8–11].

**Table 1.** Glossary of mechanical factors and mechanobiology terms.

Mechanical Factors	Description
**Mechanical Forces**	
*Stiffness*	The resistance of a material or substrate to deformation when a force is applied; commonly quantified by the Young’s modulus.
*Viscoelasticity*	A material property describing time-dependent deformation that combines both elastic (recoverable) and viscous (energy-dissipating) responses to stress.
*Topography*	The physical surface features of a material (e.g., grooves, ridges, pores) that influence cell alignment, adhesion, and function.
*Confinement*	Physical restriction of cellular space that alters cell morphology, migration, and nuclear deformation.
*Spatial Constraints (Geometric Cues)*	Physical limitations or defined geometries in the cellular environment (e.g., microchannels, micropatterns) that restrict cell shape, spreading, and movement.
*Shear Stress*	A stress component acting parallel to a surface, often generated by fluid flow across cells or tissues.
*Tensile Stress*	Stress applied that pulls or stretches a material along its length.
*Compressive Stress*	Stress applied that pushes or squeezes a material, reducing its volume or length.
*Hydrostatic Pressure*	Pressure exerted by a fluid at equilibrium on surrounding structures.
**Mechanical Properties**	
*Stress*	The internal force per unit area within a material generated in response to an applied force.
*Strain*	The relative deformation of a material caused by stress, expressed as the change in length or shape relative to the original state.
*Strain Rate*	The rate at which deformation occurs in a material over time.
*Stress Relaxation*	The gradual decrease in stress under constant strain due to viscoelastic behavior.
*Creep*	Time-dependent increase in strain under a constant applied stress.
*Young’s Modulus (Elastic Modulus)*	A quantitative measure of stiffness defined as the ratio of stress to strain in the linear elastic region of deformation.
*Elasticity*	The ability of a material to return to its original shape after deformation once the applied force is removed.
*Viscosity*	The resistance of a material or fluid to flow, reflecting internal friction during deformation.
*Substrate Rigidity*	A mechanical property of resistance to bending, but it is often used interchangeably with stiffness when describing cell adhesive substrates.
**Cell Mechanobiology**	
*Mechanotransduction*	The process by which cells sense mechanical cues and convert them into biochemical signals that regulate cellular behavior.
*Matrix Remodeling*	The dynamic process by which cells modify their surrounding extracellular matrix through degradation, synthesis, or mechanical reorganization.
*Cell Contractility (Actomyosin Tension)*	Forces generated internally by cells through actin – myosin cytoskeletal interactions, enabling cells to exert tension on their surroundings and regulate shape, adhesion, and signaling.
*Traction Forces*	Subcellular forces exerted by cells on their surrounding substrate or matrix through cytoskeletal contraction and adhesion complexes.
*Cell – Cell Contact / Intercellular Forces*	Mechanical interactions between neighboring cells mediated by adhesion molecules (e.g., cadherins) that transmit forces and regulate tissue organization and signaling.

Mechanotransduction is the process by which cells sense these mechanical signals and convert them into structural and biochemical responses [12,13]. This involves a structural network physically linking extracellular signals such as the ECM to the nuclear interior and the translocation of signaling molecules [14]. At cell-ECM adhesions, mechanical cues are sensed by adhesion receptors such as integrins, which connect the ECM to the cytoskeleton [15]. Forces can be transmitted through cytoskeletal filaments to the nuclear envelope, where they can alter nuclear organization and influence chromatin structure and gene expression [16,17]. In addition, cell deformation may directly impact nuclear mechanics and chromatin organization. In parallel, mechanical cues also trigger chemical signaling cascades that modify DNA and histone proteins at specific genomic loci, establishing dynamic patterns of gene activation and silencing [7,18]. The integration of mechanical signals with epigenetic regulation is a fundamental mechanism by which cells adapt their phenotype to their mechanical environment [7,18]. This recognition has shifted the perception of the nucleus from a passive genetic repository to a dynamic, mechanosensitive organelle whose architecture and function are actively shaped by its physical surroundings [19]. Understanding these processes has profound implications for cell reprogramming, disease modeling, and regenerative medicine – positioning the nucleus itself as a direct target for cell engineering.

To explore and understand these phenomena, numerous technologies have been developed to manipulate the mechanical inputs acting on the nucleus and to characterize the resulting outputs across cellular and molecular scales. These include biomaterials, biomedical devices, super-resolution imaging, single-cell epigenomics, and computational bioinformatics pipelines. Such tools are revealing the mechanisms of nuclear mechanotransduction and enabling the controlled modulation of chromatin organization and epigenetic states. In this review, we examine nuclear mechanotransduction from a technological perspective, highlighting advances in engineered mechanical environments, measurement platforms, and integrative approaches that together open new avenues for understanding and controlling cell fate changes through mechanical modulation of the nucleus.

### Matrix engineering for nucleus modulation

Experimental investigation of mechanotransduction pathways requires precise application of controlled mechanical stimuli. Physical manipulation technologies enable researchers to systematically modulate mechanical cues and observe resulting changes in nuclear architecture and transcriptomic and epigenetic states, establishing causal relationships between specific mechanical inputs and downstream responses. The design of biomaterials with tunable mechanical properties represents a foundational approach for studying mechanotransduction in physiologically relevant contexts. As our understanding of tissue mechanics advances, the importance of recreating appropriate mechanical microenvironments has become increasingly apparent [20,21]. For instance, recent cancer research has demonstrated how the mechanical properties of tumor cells profoundly influence malignant progression, drug sensitivity, and T cell killing [22–25], necessitating accurate mechanical modeling when investigating cancer epigenetics. Similarly, studies of cardiovascular mechanobiology require biomaterials that can recapitulate the ever-developing models of native vasculature [26,27] to properly examine how hemodynamic forces influence cell phenotypes and ECM remodeling [28]. To recapitulate *in vivo* environments in cell culture models, tunable biomaterial platforms have been developed with customizable elasticity, viscosity, and other biophysical properties [2,29,30]. Furthermore, biomaterials can be functionalized with molecules and proteins to direct cell behavior and improve biomimetic capabilities [31–33]. Another recent development in this field has been the creation of dynamic biomaterials which can change properties in response to stimuli, cell behavior, or over time [34–37]. Given emerging evidence that ECM structure, composition, and mechanical properties may be dynamic in development and disease [22,38] these biomaterials present an opportunity to further study the resulting mechanotransduction *in vitro*.

**Figure 1.**
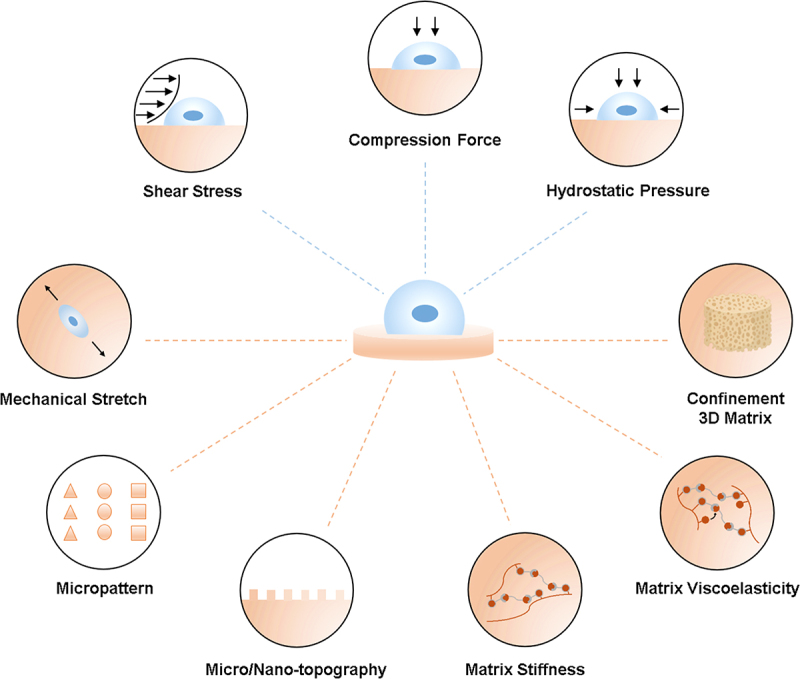
Engineering mechanical cues in the cellular microenvironment to modulate nuclear structure and function. Mechanical forces, geometry, topography, confinement, and ECM mechanical properties can be engineered to influence nuclear mechanotransduction and epigenetic regulation.

Engineering the ECM provides a powerful means to control the extracellular mechanical inputs that are transmitted into the nucleus. Through variations in stiffness, viscoelasticity, geometry, confinement, topography, and dynamic mechanical loadings, matrix-based systems modulate cytoskeletal tension, nuclear shape, and chromatin structure. This section outlines how different types of matrix engineering influence nuclear mechanotransduction and epigenetic regulation (Figure 1 and Table 2).

**Table 2.** Emerging technologies to engineer and study nuclear mechanotransduction.

Broad Method	Sub-techniques	Description
**Physical manipulation**		
Biomaterials	Tunable, dynamic, functionalized	Customized cell substrates to mimic different aspects of the *in vivo* environment
Micropatterning (lithography)	Stamp, Photo, Pen	Methods for creating (potentially functionalized) surface patterns on cell substrates
Micropatterning (3D printing)	Nozzle-based, Light-based	Depositing or solidifying material to create complex substrates and patterns
Cell-stretching devices	Pneumatic, Motor, Uniaxial, Biaxial, Equibiaxial, Cyclic	Platforms for (potentially continuous) biomimetic substrate stretching
Fluid shear stress devices/platforms	Parallel-plate flow chamber, Cone-and-plate, Orbital shaker, Microfluidic	Platforms for biomimetic fluid flow over cells
Cavitation bubbles	Needle-induced, Acoustic-induced, Laser-induced, Drop tower	Technique to generate instantaneous, collapsing bubbles for precise mechanotransduction events
**Nuclear imaging**		
Molecular force sensors	stFRET, STReTCh, ForceChrono	Uses conformational changes in fluorescently labeled proteins or DNA to visualize mechanical forces in cells
SMLM (stochastic)	STORM, dSTORM, OligoSTORM, PALM, iPALM, Deep-PALM	Superresolution microscopy technique using temporary, random fluorescent activation
SMLM (sequential)	ORCA	Superresolution microscopy to evaluate the 3D geometry of a specific locus
SIM	3D-SIM, DL 3D-SIM	Microscopy technique which leverages patterned light and deconvolution to achieve high resolution
CRISPR-based (repetitive)	CRISPR-Sirius, Casilio imaging, fCRISPR	Uses deactivated Cas units and custom designed guide RNAs to highlight repetitive DNA sequences
CRISPR-based (non-repetitive)	CRISPR FISHer, CRISPRdelight	Uses deactivated Cas units and custom designed guide RNAs to highlight target sequences
**Sequencing and computational techniques**		
Chromosome conformation technologies	3C, 4C, 5C, Hi-C, ChIA-PET, scHi-C	Captures chromatin contact and architecture information
Methylation sequencing	RRBS, scRRBS, LR-seq, EM-seq, etc.	Detects DNA methylation modifications
Transcriptome sequencing	RNA-seq, scRNA-seq, spatial, etc.	Analyzes RNA transcripts to measure gene expression
Transcriptome inference tools	CellChat, CellPhoneDB, NicheNet, etc.	Use ligand-receptor databases to infer cell-cell and cell-ECM communication
Chromatin accessibility	ATAC-seq, scATAC-seq, spatial, LR-seq, etc.	Combines next-generation sequencing with specialized enzymes and reagents to measure chromatin accessibility
Multiomics and integration packages	MUDI, scMI, SEE	Computational methods to integrate sequencing methods for more comprehensive analyses

### Micropatterning and topography on nuclear deformation and epigenome

For more spatially defined control of the cellular microenvironment, micropatterning techniques have been widely used to control cell adhesion geometry, cell morphology, and cell-cell interactions [39] which can influence cytoskeletal organization, and nuclear and chromatin arrangement. Stamp lithography (microcontact printing) employs elastomeric stamps to create defined patterns on a substrate [40–44] enabling precise control over cell spreading and resulting tension states. Photolithography offers enhanced spatial resolution through light-controlled patterning [42,43], with recent innovations achieving nanoscale features through manipulation of interference patterns [45]. Even finer control comes from pen lithography techniques, which adapt atomic force microscopy (AFM) technology to ‘write’ nanoscale patterns with extraordinary precision [46,47], and three-dimensional (3D) bioprinting, which use needle-deposited or light-activated substrates to create intricate designs [48]. By systematically manipulating cell shape and adhesion geometry, these micropatterning approaches have revealed how spatial constraints on adhesion translate to nuclear deformation and chromatin reorganization [43,49–52]. For example, cell geometric constraints can alter cytoskeletal contractility and organization to regulate nuclear morphology, nuclear lamina levels and chromatin condensation [52].

In addition to micropatterning, biomaterials can be designed to present topographical cues that mimic the complex topographic structures of the ECM. Various fabrication methods, including soft lithography, chemical etching, electrospinning and 3D printing, can be utilized to engineer scaffolds with nano- or micron-sized features in a controllable and reproducible fashion [53–55], thereby providing researchers a platform to study how cells respond to these physical signals. Topographical cues, such as roughness and the size, shape and arrangement of surface patterns, have been shown to influence cell adhesion, migration, differentiation and gene expression in response to contact guidance [9,50,53,56–62]. More recent evidence indicates that surface topography can regulate nuclear morphology, chromatin accessibility and the epigenome, thereby facilitating cell fate transitions [18,63–74]. For example, microgrooves and aligned nanofibers can induce changes in histone H3 acetylation and methylation that promote the reprogramming of fibroblasts into induced pluripotent stem cells [63]. Additionally, in macrophages it was shown that micropattern-induced cell elongation suppresses inflammatory signaling by modulating a cytoskeleton-dependent Src – p300 pathway that regulates histone H3 acetylation [75]. Similarly, it has been reported that micropillar pattern-induced nuclear deformation alters nuclear architecture, chromatin conformation and transcriptional responsiveness to differentiation cues, thereby facilitating the differentiation of mesenchymal stem cells into the osteogenic lineage *in vitro* and promoting bone regeneration *in vivo* [71]. These findings highlight how the integration of topographical cues in implantable biomaterials could potentially be utilized to enhance tissue regeneration via chromatin reprogramming.

### Cellular confinement, 3D lithography, and bioprinting of matrix

Devices engineered to impose a physical confinement on cells have been utilized to investigate the effects of confinement that cells may experience *in vivo*, particularly cell migration through tight spaces [76,77]. The confinement of adherent cells, which can be studied in either a 2D or 3D context [78,79] can induce cellular and nuclear deformation, which can lead to changes in nuclear shape, volume, and lamina organization, thereby resulting in nuclear envelope rupture and DNA damage in cases of extreme nuclear deformation [80] or potentially affect gene expression [81,82] and cellular functions such as migration [83,84] spreading [83] and differentiation [82,85]. For example, microfluidic devices have been developed to monitor and quantify nuclear deformation in real-time [86–89] in particular during cell migration, providing insights into nuclear mechanics, structure, and their impact on cell behavior and disease processes. These microfluidic devices differ from microfluidic systems in that there is only local space confinement and no microfluidic perfusion in the device. More recently, mechanically-induced confinement has been shown to elicit changes in chromatin organization and the epigenetic state that alter downstream cellular processes such as cell differentiation [82,85,90], offering insights into mechano-epigenetic mechanisms that regulate stem cell differentiation.

Bio-metamaterials patterned via 3D multiphoton lithography allow researchers to engineer mechanical responses not attainable in bulk materials, including programmable Poisson’s ratios (e.g., negative values in auxetics), independently tunable elastic parameters (e.g., Young’s modulus vs. shear modulus), highly anisotropic and spatially defined mechanical fields, geometry-driven nonlinear responses, dynamic deformability for real-time force sensing, and scaffold unit cells at sub-cellular resolution. In particular, the introduction of IP-polydimethylsiloxane (IP-PDMS) in 2021 unlocked the ability to print soft, deformable metamaterials via multiphoton 3D lithography [91]. This advance enabled the creation of suspended elastic bio-metamaterial nets that could mechanically tune cell shape, traction forces, and YAP signaling in human MSCs, serving as a proof-of-concept platform for cell mechanoregulation [92].

Advances in 3D printing have allowed the fabrication of scaffolds with controlled architecture, anisotropy, and mechanical gradients. More specifically, additive manufacturing strategies such as stereolithography and direct-write can produce matrices with tunable porosity, geometry, and spatially heterogeneous stiffness [93]. For example, fused deposition modeling was utilized to print biphasic constructs containing a porous cartilage-like region over a dense bone-like substrate to replicate the stiffness of native osteochondral tissue. This matrix was used to regulate region-specific cell differentiation and ECM deposition, suggesting that spatial variation in matrix rigidity can direct chromatin organization and tissue-specific nuclear morphology [94]. Similarly, a filamented-light (FLight) fabrication technique was utilized to introduce fibrillar structures within 3D hydrogel microgels, creating an anisotropic mechanical environment at the cellular level [95]. It was found that these topographical features guide the orientation of encapsulated C2C12 myoblasts along the axis of the fibers, thereby enhancing cytoskeletal alignment and nuclear elongation by imposing anisotropic tension on the nucleus. Findings from these studies demonstrate that 3D printing can be utilized to introduce macro and microscale features into matrices, which then impose specific mechanical cues on the nucleus that drive nuclear deformation.

Intravital 3D bioprinting harnesses multiphoton excitation to fabricate cell-laden hydrogels with micrometer-scale resolution directly within intact tissues. By introducing nontoxic cell-laden photosensitive polymer hydrogels that can be bioprinted within tissue, it has been demonstrated that there can be precise spatial modulation of photopolymerized hydrogels *in vivo* without disruption of the surrounding anatomy [96]. *In vivo* application revealed that topological confinement and anisotropic mechanical cues alone are sufficient to direct stem cell alignment, promote myofiber formation, and induce nuclear reorganization, demonstrating that engineered mechanical microenvironments can drive cell fate decisions and tissue morphogenesis under fully physiological conditions.

### Effects of substrate stiffness and viscoelasticity on epigenome modulation

Hydrogels have emerged as powerful artificial cell culture systems in mechanobiological studies, as their high water content and porous structure closely resemble the native ECM. Additionally, their material properties can be finely tuned through various physical and chemical strategies [97,98]. For example, polyacrylamide hydrogels are among the earliest and most widely used biomaterials in the field of mechanobiology. By adjusting the concentrations of acrylamide monomer and bis-acrylamide, as well as crosslinking conditions such as polymerization temperature and gelation time, the stiffness of these hydrogels can be precisely controlled, ranging from below 1 kPa, mimicking soft tissues like brain, to several hundreds of kilopascals, representing stiff tissues such as bone [99–101].

Other hydrogel systems utilize naturally derived polymers, including protein-based materials like collagen and polysaccharides such as alginate and hyaluronic acid, and synthetic polymers including poly(ethylene glycol) (PEG), vinyl polymers and polypeptides [102]. Natural protein-based hydrogels mimic native ECM biochemistry but suffer from batch-to-batch variation and limited tunability due to their heterogeneous composition. Naturally derived polysaccharides offer greater control over mechanical properties, although the batch variation remains a concern. In contrast, hydrogels made from synthetic polymers exhibit superior flexibility in tailoring material properties. A broad range of functional groups can be synthesized to the polymer backbones to provide desirable functionalities, such as cell adhesiveness, biodegradability, and independently tunable mechanical properties. However, the potential cytotoxicity of chemical reagents, residual crosslinkers, and reaction byproducts must be carefully evaluated, particularly for their applications involving 3D or long-term cell culture. It is also important to use appropriate techniques to measure viscoelastic materials [30].

Over the past few decades, matrix stiffness has been widely recognized as a key regulator of numerous cellular processes, including proliferation, migration, and differentiation. More recent evidence indicates that matrix stiffness can regulate these processes by altering the epigenetic state of cells, including the activity of histone modifying enzymes and DNA methylation [103–117]. In turn, this leads to chromatin remodeling in cells that can affect gene expression and cell function, where aberrations in chromatin organization in response to matrix stiffness can potentially lead to age-related or diseased phenotypes. A recent study demonstrated that matrix stiffness biphasically modulates histone acetylation and chromatin accessibility to enhance cell reprogramming, with an intermediate stiffness of 20 kPa yielding the most optimal results [106]. This was found to be dependent on the shuttling of histone acetyltransferase into the nucleus by actin cotransporters (i.e. G-actin and cofilin), which was mediated by matrix stiffness and particularly limited on soft matrices.

However, native ECMs exhibit a much broader set of mechanical behaviors, including viscoelasticity, plasticity, and other non-linear responses [2] which cannot be fully captured by linearly elastic models. To address this, dynamic crosslinking strategies have been developed to better mimic the time-dependent properties of native ECMs [2,118,119]. Unlike permanent covalent crosslinking that forms stable and irreversible chemical bonds, dynamic covalent chemistries and physical interactions, such as ionic crosslinking, host-guest interactions, hydrogen bonding, enable reversible and weaker interactions between functional moieties or polymer chains in the hydrogel network, allowing for stress relaxation, energy dissipation, and other non-linear mechanical behaviors [120]. Moreover, incorporating dynamic crosslinking into traditionally elastic systems like polyacrylamides has led to the construction of viscoelastic hydrogels [121,122] expanding their application in mechanobiology by more closely mimicking the complex mechanics of native tissues.

With the development of viscoelastic materials, matrix viscoelasticity has been pointed to play critical roles in regulating both cellular processes and tissue-level behaviors [120,123–126]. However, the underlying mechanisms, especially whether this involves epigenetic regulation, remain largely unexplored. A recent study demonstrated that fibroblasts cultured on viscoelastic substrates exhibited reduced lamin A/C expression, lower chromatin compaction, increased expression of euchromatin marks, and locally enhanced chromatin accessibility in genomic regions associated with neuronal genes [127]. Together, these epigenetic changes facilitated more efficient cellular reprogramming of fibroblasts into neurons [127] suggesting that matrix viscoelasticity may influence cell fate decisions through modulation of the epigenome. These findings highlight an emerging role of ECM viscoelasticity in shaping chromatin architecture, opening new directions for understanding how cells integrate complex biophysical cues into long-term genetic and functional changes, with significant implications for development, disease modeling, and regenerative medicine.

### Dynamic mechanical loading effects on the nucleus

For larger-scale changes to the cell environment, cell-stretching devices can provide dynamic mechanical stimulation, mimicking the forces experienced by cells in many physiological contexts. These systems vary in their actuation mechanisms (pneumatic or motor-driven) and in their directional capabilities [128–131] (uniaxial, biaxial, or equibiaxial deformation). Advanced systems now incorporate cyclic loading protocols that replicate physiological rhythms such as breathing cycles or cardiac contractions, and feature microscope compatibility for real-time visualization of nuclear responses [130]. Mechanical stretch can alter many aspects of cellular function and behavior, including proliferation, migration and differentiation, by regulating the cytoskeleton, nucleus and epigenome [132–137]. Similarly, compressive forces [138,139] and hydrostatic pressure [140] have been shown to induce chromatin reorganization and epigenetic changes that alter cell fate and function.

Fluid shear stress platforms present another dynamic cell culture option to reproduce the hemodynamic forces experienced by vascular cells and numerous other cell types exposed to fluid flow *in vivo*. These systems span diverse designs, from parallel-plate flow chambers offering uniform shear profiles to cone-and-plate devices generating rotational flows, orbital shakers providing high-throughput capabilities, and microfluidic platforms enabling precise spatiotemporal control of flow patterns [141–143]. These technologies have been instrumental in revealing how fluid forces activate mechanosensitive pathways that ultimately influence nuclear organization and gene expression profiles, with relevance to vascular biology and epithelial mechanosensing.

In addition, cavitation bubble techniques can deliver localized, transient mechanical perturbations at cellular and subcellular scales. Methods including needle-induced [144,145] acoustic-induced [144,146] laser-induced [147,148] and drop tower [144,149] approaches generate precise mechanical disruptions that reveal threshold responses in nuclear mechanics and rapid mechanotransduction events. These techniques have proven particularly valuable for studying the mechanical forces present in acute events such as blunt force brain trauma [150,151] and vascular injuries [146].

## Nuclear Characterization by imaging

In addition to ECM, various platforms have been developed to characterize the deformation and mechanical properties of the cell and nucleus (Table 3). These physical manipulation technologies form a comprehensive toolkit for investigating how specific mechanical stimuli propagate from the cell surface to the nucleus. Physical techniques that apply defined forces or constraints therefore provide quantitative mechanical readouts, such as stiffness, viscoelastic response, deformability, and anisotropy that serve as intermediate phenotypes linking mechanical inputs to downstream nuclear and chromatin regulation. By systematically controlling mechanical parameters, researchers can establish direct causal links between defined mechanical inputs and resulting nuclear responses, advancing our mechanistic understanding of nuclear mechanotransduction.

**Table 3.** Techniques for inducing cell and nuclear deformation and measuring mechanical properties.

Technique	Force Application Mode	Mechanical Readout	Throughput	Key Cellular Structures Probed
Micropipette Aspiration	Pressure-driven suction	Stiffness, viscoelasticity, interfacial tension	Low but can be high when integrated into microfluidic devices [152–154]	Cell, nucleus, lamin, chromatin density [155,156]
AFM	Local indentation force	ECM stiffness [157,158], cell mechanical properties [159,160], nuclear stiffness, and viscoelasticity [161,162]	Low	Cell, nucleus
Optical Tweezers	Point force via trapped bead	Viscoelasticity in cells and ECM [163], deformation and stiffness in cells and organelles, protein conformation on the cell surface [164]	Low	Cell, chromatin, protein complexes
Magnetic Tweezers	Directional force on bead	Local and intracellular viscoelasticity [165–170]	Low – medium	Cell, chromatin – lamina coupling [171]
Microfluidic Platforms	Confinement and fluid flow	Deformability [172–176], transit time	High	Cell, nucleus, lamin, chromosome [177–179]

Across platforms, mechanical measurements rely on a shared physical principle. A defined force, pressure, or geometric constraint is applied to a cell or nucleus, and the resulting deformation, displacement, or relaxation dynamics are quantified. By relating applied forces to measured deformation through analytical or computational frameworks, such as force – displacement relationships, creep or stress – relaxation analyses, or phenomenological viscoelastic models, these approaches yield effective mechanical descriptors including elastic or viscoelastic moduli, apparent stiffness, deformability indices, and directional anisotropy [180]. Importantly, these quantities reflect the combined mechanical contributions of multiple cellular and nuclear components rather than isolated molecular elements, and their values depend on loading geometry, boundary conditions, and timescale. As such, different techniques provide complementary and context-dependent mechanical readouts that together define the mechanical state of the cell and nucleus. Table 3 compares some methods commonly used for mechanical property measurements and to induce cellular and nuclear deformation – micropipette aspiration [181–186], atomic force microscopy (AFM) [187–189], optical tweezers [190], magnetic tweezers [191], and microfluidic platforms [192–195].

**Figure 2.**
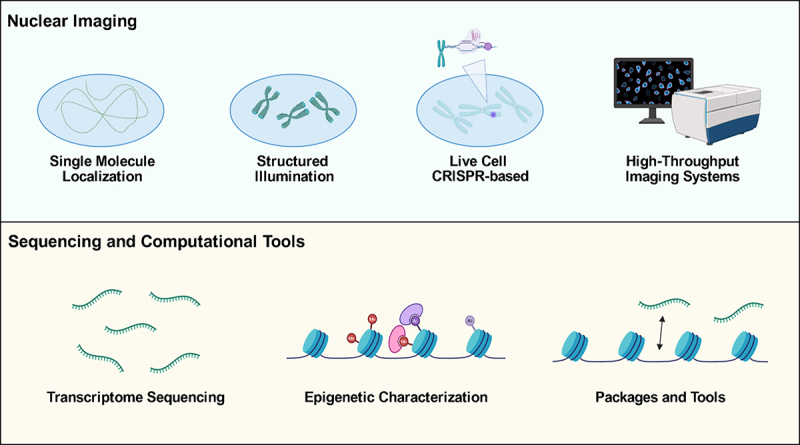
Nuclear imaging and sequencing technologies for studying mechanotransduction. Nuclear imaging approaches allow for visualization of nuclear structural changes (top), whereas sequencing and computational methods provide comprehensive, genome-wide perspectives of cell behavior, connecting physical forces to specific changes in transcription, chromatin accessibility, and three-dimensional genome organization (bottom).

The translation of mechanical signals from the extracellular space ultimately manifests as changes in nuclear structure and chromatin organization. Visualizing these nuclear responses often requires specialized imaging approaches capable of resolving structures beyond the diffraction limit of conventional microscopy. Recent advances in super-resolution and live-cell imaging have revolutionized our ability to observe mechanically induced changes in nuclear architecture across multiple scales (Figure 2 and Table 2).

### Molecular sensors for force measurement

In addition to probing techniques, there are also fluorescent mechanical sensors that can be embedded into biological systems. These molecular force sensors (MFS) take advantage of conformational changes in nucleic acids or proteins to activate fluorescent labels for imaging of piconewton-scale forces [196]. Some of these techniques, including the first MFS described in 2008 [197] use fluorescence resonance energy transfer (FRET) as their molecular strain reporter [198]. The STReTCh [199] system modifies a known mechanosensitive protein to present fluorescent label binding sites in certain conformations. The recently reported ForceChrono [200] system instead uses a dual hairpin DNA probe to activate reporters at two different force thresholds for additional insights into underlying mechanical forces in cells. These novel methods are largely designed to use existing microscopy systems, making them relatively accessible for characterization research.

### Super-resolution microscopy

Super-resolution microscopy techniques have overcome the fundamental diffraction barrier that previously limited our view of nuclear structures. Scanning confocal microscopes equipped with sensitive area detectors can achieve higher resolutions while maintaining high imaging speeds, enabling live-cell super-resolution imaging of chromatin displacement in real time [201]. Stimulated emission depletion (STED) microscopy employs a scanning illumination strategy [202] to achieve resolution below 50 nm, revealing previously invisible details of chromatin organization [203] and enabling measurement of live cell responses to mechanical perturbation [204]. Single molecule localization microscopy (SMLM) approaches, including STORM [205] and PALM [206] have become essential tools for nuclear imaging since their introduction in 2006 [207–209]. These techniques activate sparse subsets of fluorophores in each frame, enabling precise localization of individual molecules and subsequent reconstruction of super-resolved images [210]. SMLM has been utilized to track localization of a nuclear envelope mechanotransducing protein in response to cell-shaping micropatterns [211], visualize chromatin organization following mechanical stimulation [212], and track a key nuclear lamina protein [213]. These advances have transformed our understanding of mechanically induced chromatin reorganization by revealing nanoscale structural changes previously invisible to researchers.

For broader visualization of chromatin dynamics, structured illumination microscopy (SIM) provides a powerful approach for observing global changes in nuclear architecture. By illuminating samples with patterned light, SIM encodes high-frequency information that computational reconstruction transforms into super-resolved images [214]. Researchers have utilized SIM to study interactions between cytoskeletal fibers and the nuclear lamina, finding that nuclear indentation initiated segregated chromatin condensation domains [215]. This technique also has the potential to reveal large-scale chromatin movements and compartmentalization changes in response to mechanical forces, while quantitative analysis could enable objective comparison of chromatin distribution and compaction between experimental conditions [210,216]. Recent integration of deep learning approaches has further enhanced SIM resolution to approximately 120 nm isotropically in three dimensions [217] expanding its utility for future nuclear mechanotransduction studies.

### Chromatin labeling techniques

The power of traditional and super-resolution microscopy depends on advanced DNA labeling strategies. Fluorescence in situ hybridization (FISH) has been a prominent option for chromosome labeling since its inception in the late 1970s [218]. By employing fluorescently labeled probes complementary to target DNA sequences, FISH has enabled researchers to explore chromosome localization in response to mechanical perturbations of the nucleus [51]. Oligopaints, bioinformatically designed probe sets for FISH reported in 2012 [219], have undergone continuous refinement for compatibility with various super-resolution approaches, including specialized variants for STORM (OligoSTORM) and DNA-PAINT [220] (OligoDNA-PAINT). Perhaps most significantly, the integration of sequential oligopaint labeling with super-resolution imaging has enabled comprehensive visualization of chromatin structure in situ [221,222] and has recently been used to track the localization of key pluripotency genes during nuclear mechanotransduction associated with stem cell differentiation [223]. The optical reconstruction of chromatin architecture [224] (ORCA) approach, which employs sequential imaging to trace chromatin fibers, has provided unprecedented views of three-dimensional genome organization at the target loci. Furthermore, the identification of a fluorescent dye that stains DNA has allowed for visualization of chromatin in situ using ChromEM tomography, demonstrating the structural compaction and high packing densities of chromatin chains [225].

While fixed-cell super-resolution provides structural snapshots, understanding the dynamic nature of mechanotransduction often requires live-cell approaches. CRISPR-based imaging technologies have emerged as powerful tools for tracking specific genomic loci in living cells with the potential to provide real-time visualization of mechanically-induced chromatin changes using catalytically inactive Cas proteins to target fluorescent markers to specific DNA sequences [226,227]. The CRISPR-Sirius [228] system offers enhanced brightness and stability for robust live-cell tracking, while CRISPR FISHer [229] and CRISPRdelight [230] extend these capabilities to non-repetitive genomic regions. The recently developed fluorogenic CRISPR [231] (fCRISPR) system further enhances signal-to-noise ratio, potentially enabling more sensitive detection of chromatin dynamics in response to mechanical stimulation. Although not yet widely adopted, these live-cell approaches could unravel the temporal dimension of nuclear mechanotransduction, uncovering how mechanical forces trigger immediate chromatin reorganization that precedes subsequent transcriptional and epigenetic changes.

### High-throughput imaging systems

While super-resolution techniques provide nanometer-scale insight into nuclear structure and dynamics, they are often limited by throughput, constraining the ability to capture phenotypic heterogeneity or identify subtle mechanical phenotypes across large cell populations. To overcome this, emerging high-throughput and scalable 3D imaging systems provide population-level profiling with single-cell resolution. Light-field flow cytometry enables fast, near-diffraction limited 3D imaging of ~ 5000 cells/s, capturing nuclear morphology. Recent work demonstrated its use for profiling nuclear condensation and fragmentation during apoptosis, revealing nuclear alterations at scale [232]. Arrayed readout formats have also been explored to image nuclear morphological alterations in live non-adherent cells at scale with isotropic, near-diffraction-limit resolution, using picoliter droplet encapsulation to fluidically isolate single cells from each other and induce rotation via microvortices for 360° observation [233]. The ability to observe populations of cells in their suspended state is biologically meaningful, as non-adherent cells exhibit distinct cytoskeletal architectures, including dynamic ruffling and isotropic actin networks, which are rapidly remodeled upon adhesion [234]. Moreover, recent developments in imaging capabilities allow for the tracking of cellular and nuclear volume dynamics during confined migration [89], revealing the interconnectedness of nuclear volume and surface area during adaptation to confinement and providing novel insights into nuclear morphology regulation in a physiological environment.

High-content and throughput image-based phenotypic profiling methods like PERISCOPE integrate CRISPR-Cas9 knockouts (>20,000 genes) with multiplexed cell painting techniques to capture subcellular morphology, including nuclear structure, across > 30 million single cells [235]. This method extracts thousands of quantitative nuclear ‘fingerprint’-like features (size, shape, texture and intensity [236]) that enable large-scale exploration of how gene function modulates nuclear architecture, which could offer valuable insight into the downstream consequences of mechanotransduction on chromatin organization. Other applications of large-scale image-based profiling include the OpenCell project which combines endogenous protein tagging with interactome mapping and microscopy to reveal systems-level insights into the spatial organization and subcellular localization of the human proteome [237], including within the nucleoplasm and the nuclear envelope. Thus, this large-scale spatial proteomics effort offers a useful reference point for understanding how mechanical cues might reshape nuclear organization by altering protein localization dynamics. More recently, the seamless integration of image-based phenotyping with deep learning techniques such as Prediction of Unseen Proteins’ Subcellular localization (PUPS) have enabled accurate, single-cell inference of nuclear and cytoplasmic localization for unmeasured proteins across unmeasured cell types though combination of protein sequence embeddings with structural context from cellular stains. Although adoption of these image-based phenotypic profiling methods (e.g. PERIOSCOPE, OpenCell and PUPS) for mechanobiology contexts remains limited, these methods exemplify the growing synergy between high-content microscopy and data-driven computational approaches, offering scalable frameworks to infer how genetic or mechanical perturbations may reconfigure nuclear architecture and localization landscapes at single-cell resolution.

### Quantitative imaging of nuclear mechanics

Nuclear morphology and structure are typically measured using the periphery of the nucleus. This approach, which focuses on geometric descriptors, such as volume, elongation, or principal dimensions, is suitable for large-scale population analyses. However, it reduces internal chromatin heterogeneity and deformation into bulk descriptors, which obscures the spatially resolved mechanical state of the cell.

Beyond envelope-based nuclear morphology measurements, quantitative nuclear mechanobiology necessitates imaging methods capable of resolving subcellular displacements and linking image-derived observables to mechanical descriptors. Perturbation-based approaches, such as optical elastography [238–240] and deformation mapping [241,242] have enabled the computational reconstruction of strain fields and effective stiffness across multiple spatial scales under defined mechanical loads. Early in situ demonstrations of deformation-based methods showed that physiologically relevant tissue-scale loading can produce heterogeneous intranuclear strain distributions, with local strain amplification reaching higher values than the applied macroscopic deformation [239].

In this framework, a key challenge is the conversion of microscopy-derived displacement fields into physically meaningful intracellular strain and stress measurements. Deformation microscopy (DM) tackles this challenge by combining high-resolution confocal z-stacks with automated hyperelastic warping and deformable image registration to compute three-dimensional intranuclear displacement fields before and after a controlled mechanical perturbation [241]. By removing rigid-body motion (i.e. rotations and translations), local strain tensors, including hydrostatic, deviatoric, and principal strains can be computed, enabling a spatial mapping of intranuclear deformation.

The technique has been successfully used to capture intranuclear heterogeneity associated with distinct strain hotspots at specific chromatin domains, at heterochromatin and euchromatin regions [238,240], revealing that mechanical deformation is not uniformly distributed, but instead is spatially patterned in a manner that reflects underlying chromatin organization. It has further been shown that DM can map biophysical interactions incoming from the stiffness of the substrate and cytoskeletal organization, revealing that nuclear strain transmission is not solely a passive mechanical consequence but is actively modulated by nucleocytoskeletal coupling. Moreover, in cardiomyocytes, disruption of the LINC complex via dominant-negative KASH constructs or nesprin-3 knockdown significantly reduced tensile hydrostatic strain within the nucleus during contraction, despite comparable cellular deformation [241], thus suggesting that nuclear lamina and LINC complexes are not merely structural components, but critical regulators of force propagation.

#### Non-contact methods

Label-free optical approaches have enabled direct mechanical interrogation of nuclear compartments without external perturbation. Among these, Brillouin microscopy, which probes the longitudinal modulus through light-acoustic phonon interactions, has provided one of the first non-contact methods to spatially resolve nuclear stiffness [243]. Brillouin microscopy is based on inelastic light scattering from thermally excited acoustic phonons, in which the measured Brillouin frequency shift (BFS, $\Omega_{B}$) reflects the local longitudinal elastic modulus at GHz frequencies [244]. In hydrated biological materials, including nuclei for example, the BFS typically falls in the 5–8 GHz range and reports on the longitudinal storage modulus ($M{\;}^{\prime }\propto \Omega_{B}^{2}$), while the linewidth (Γ) reflects viscous dissipation. Using submicron-resolved confocal Brillouin imaging, Antonacci and Braakman showed that distinct nuclear compartments have different longitudinal moduli, with nucleoli being stiffer than both the surrounding nucleoplasm and cytoplasm [245]. Critically, they demonstrated that cytoskeletal disruption via latruculin-A selectively reduced cytoplasmic stiffness, while largely preserving nucleolar mechanics, indicating that nuclear substructures possess intrinsic mechanical properties that are not uniquely dictated by cortical actin. In another study, chromatin decondensation via histone deacetylase inhibition resulted in measurable reductions in nuclear Brillouin shift, directly linking epigenetic chromatin state to mechanical phenotype [246]. These results therefore underscored that nuclear longitudinal modulus is sensitive to chromatin packing density and hydration.

Extending this framework to population-level measurements, Brillouin flow cytometry was developed to mechanically phenotype nuclei in liquid suspension without physical deformation [247]. In their system, cells were probed individually within a microfluidic channel, enabling mechanical phenotyping at throughputs of ~100–200 cells per hour. Although this metric is significantly lower than those of deformability cytometry [172] (~10^3^-10^4^ cells/min), it provides a fundamentally different metric related to high frequency longitudinal modulus, without applying external mechanical stress.

Recent pulsed Brillouin implementations have reduced photo dose by up to two orders of magnitude while maintaining MHz-level spectral precision, enabling high-resolution viscoelastic mapping in living cells with improved signal-to-noise ratios and sub-second acquisition times [248]. Furthermore, fast live Brillouin modalities now allow the monitoring of mechanical responses in the second scale, revealing compartment-specific nuclear dynamics under acute perturbation [249]. Using this method, nucleoli were found to consistently exhibit higher baseline Brillouin shifts than nucleoplasm and cytoplasm, confirming intrinsic mechanical heterogeneity, while cytoskeletal disruption induces rapid (seconds-scale) reductions in Brillouin shift predominantly in cytoplasm and nucleoplasm, with comparatively smaller effects in nucleoli.

Complementary to deformation-based approaches, recent advances in live-cell correlative lattice light sheet microscopy (LLSM) combined with 3D single-molecule tracking have enabled the inference of nuclear material properties directly from endogenous chromatin fluctuations [250]. Rather than requiring the application or presence of external perturbations, this framework can extract diffusion coefficients and anomalous exponents from intrinsic nucleosome dynamics to probe local viscoelastic behavior within the nucleus and the interchromatin space. For example, an inverse relationship between nucleosome mobility and chromatin packing was demonstrated by tracking Halo-tagged H2B and measuring nucleosome mean square displacement and chromatin density [250]. Super-resolution packing measurements, combined with polymer modeling, further showed that passive polymer behavior alone does not fully account for nucleosome dynamics in sparsely packed chromatin [250]. Moreover, inhibition of RNA polymerase II reduced this mismatch, indicating that transcriptional activity locally stabilizes chromatin and modulates its mechanical constraints. Overall, these results suggest that nuclear mechanics reflect not only chromatin density but also ongoing genomic activity.

In parallel, an imaging-based chromatin remodeling assay was developed to quantify nanoscale intranuclear structural reorganization under oxidative stress [201]. In that study, the use of high-resolution live-cell microscopy and a computational Chromatin Remodeling Index demonstrated that hyperoxia induces rapid, spatially heterogeneous chromatin compaction as opposed to uniform nuclear condensation. In addition, zone-specific analysis revealed that euchromatin domains undergo significant remodeling, while heterochromatin remains less dynamic in comparison. Importantly, pharmacological inhibition of EZH2 using GSK126 significantly attenuated both chromatin compaction and remodeling dynamics and complementary ATAC-seq profiling showed that oxidative stress reduces genome-wide chromatin accessibility, partially rescued by EZH2 inhibition. The results reinforce the idea that nuclear mechanical behavior emerges from the interaction between chromatin organization, epigenetic state, and environmental stress, extending fluctuation-based viscoelastic inference to include actively regulated structural remodeling in living cells.

Together, these advanced nuclear imaging technologies have transformed our understanding of nuclear mechanotransduction by visualizing previously invisible structures and dynamics. By spanning scales from individual nucleosomes to whole-genome organization, and from static snapshots to dynamic processes, these approaches reveal how mechanical forces reshape nuclear architecture to influence gene expression and cell fate. The continued integration of these imaging technologies with physical manipulation approaches promises even deeper insights into how mechanical signals propagate from the extracellular space to the nucleus to regulate epigenetic processes.

## Sequencing and computational techniques

While imaging approaches visualize nuclear structural changes, sequencing and computational methods can provide comprehensive, genome-wide perspectives of cell behavior (Figure 2 and Table 2). These technologies map the molecular landscape of mechanotransduction, connecting physical forces to specific changes in transcription, chromatin accessibility, and 3D genome organization.

Transcriptomic profiling offers a direct window into the functional consequences of mechanotransduction pathways, revealing how mechanical signals ultimately alter gene expression programs. RNA sequencing [251] (RNA-seq or bulk RNA-seq) approaches can enable systematic identification of gene and pathway expression changes in response to mechanical forces [252–254] and variable physical environments [255–257] by capturing and sequencing mRNA transcripts. The emergence of single-cell RNA-seq [258] (scRNA-seq) has further refined our understanding by revealing cellular heterogeneity in mechanotransduction responses [259–261], and even demonstrating how apparently uniform mechanical stimuli can trigger diverse transcriptional outcomes within cell populations [262]. For example, researchers have recently used scRNA-seq to identify distinct subpopulations with varying mechanosensitivity and differentiation capability arising from mesenchymal stem cells grown on soft or stiff substrates [263]. In addition to characterizing single cells, recent development of spatial transcriptomics [264–266] preserves crucial positional information, enabling researchers to correlate local mechanical properties, such as regional differences in substrate stiffness or micropatterned geometries. In this realm, a recent computational framework was developed which enables ‘spatial mechano-transcriptomics’ via integration of tissue imaging, segmentation, spatial transcriptomics, and a scRNA-seq cell type atlas [267]. Using this technique, researchers can infer spatially resolved tension and pressure on cells in addition to transcriptomic profiles, enabling analysis of the interplay between mechanical forces and gene expression at a single-cell level.

Epigenetic sequencing techniques probe deeper into the mechanisms by which mechanical forces influence gene regulation. ATAC-seq [268] (Assay for Transposase-Accessible Chromatin using sequencing) can reveal how mechanical stimuli alter chromatin accessibility landscapes [111,113,269,270], identifying specific genomic regions that become more open or condensed in response to physical cues. Recently, several researchers have developed protocols for single-cell [271] and spatial [272,273] ATAC-seq, which has the potential to further illustrate effects of biophysical cues on chromatin arrangement. Various methylation sequencing approaches, including reduced representation bisulfite sequencing [274] (RRBS-seq), enzymatic methylation sequencing [275] (EM-seq), and nanopore long-read sequencing [276,277] (LR-seq), can track changes in DNA modification patterns that reflect epigenetic adaptations or responses to physical environments [110,278]. However, the investigation of differential DNA methylation in response to mechanical stimulation via sequencing still remains largely unexplored. The emergence of single-cell variants [279,280] for these assays additionally enables unprecedented resolution of epigenetic heterogeneity which may be used to investigate mechanically stimulated populations. Particularly noteworthy is LR-seq, which can be modified to enable simultaneous assessment of multiple epigenetic features [281,282] (accessibility, methylation, and genomic variation) along continuous stretches of DNA, enabling future studies of how mechanical forces coordinate diverse epigenetic modifications.

For investigating higher-order chromatin organization changes, chromosome conformation capture technologies have proven invaluable. The 3C family of methods [221,283,284] (3C, 4C, 5C, Hi-C, ChIA-PET, and single-cell variants) map physical interactions between genomic regions, revealing how mechanical forces reshape the three-dimensional architecture of the genome [285–290]. As discussed in a recent review [284] these approaches (and others) could be used to investigate how mechanotransduction affects topologically associating domains (TADs), modifies chromatin compartmentalization, and establishes or disrupts specific enhancer-promoter loops. Furthermore, the ability to evaluate chromatin contacts at a genome-wide level has led to a growing interest in developing polymer models which can mechanistically translate structural snapshots into predictive physical frameworks. For instance, sequencing-informed copolymer simulations in a multi-omic study have recently quantified how heterochromatin domain boundaries shift in direct response to substrate stiffness, revealing how physical cues drive epigenetic memory [291]. Similarly, stochastic polymer mechanics models have shown that a significant portion of DNA contact frequency can be predicted purely by the physical spacing and positioning of nucleosomes between loci [203]. By integrating biophysical constraints with high-resolution sequencing, these computational methods move the field from descriptive mapping toward a causal understanding of how mechanical strain reshapes genomic function.

The growth in sequencing methods and their accessibility has necessitated sophisticated computational approaches for interpreting the expression of tens of thousands of genes. Tools specialized for signaling pathway analysis, such as CellChat [292] CellPhoneDB [293] and NicheNet [294] can help reconstruct the complex cellular communication cascades that connect mechanical stimuli to transcriptional responses [295–297]. The increasing integration of spatial transcriptomics with these tools, exemplified by CellChat’s new spatial capabilities [298], will provide contextual information crucial for understanding mechanotransduction in future studies. For researchers seeking comprehensive information on these computational resources, two recent reviews [299,300] offer valuable guidance on their applications and limitations.

Perhaps most promising is the growing capacity for multi-omic data integration, which combines different sequencing modalities to provide holistic views of mechanotransduction. Novel computational approaches such as scMI [301] enable database-independent integration of RNA-seq and ATAC-seq data through machine learning of cross-modality relationships. Tools like SEE [302] and MUDI [303] integrate chromatin conformation (scHi-C) with gene expression (scRNA-seq) data, potentially enabling researchers to learn how mechanical forces might simultaneously alter genome architecture and transcriptional outputs. Other emerging approaches, such as scGrapHiC [304] and SCRIPT [305], leverage prior models or multimodal data to extract additional information from Hi-C sequencing. Specialized tools addressing technical challenges in single-cell Hi-C data (scDEC-Hi-C [306] for clustering, BandNorm and scVI-3D [307] for normalization and de-noising) will further enhance our ability to gather meaningful insights from future complex mechanotransduction datasets.

Collectively, these sequencing and computational approaches provide the molecular resolution needed for researchers to connect mechanical stimuli to specific transcriptomic and epigenetic outcomes. By systematically mapping changes in transcription, chromatin accessibility, DNA modifications, and three-dimensional organization across the genome, these technologies may reveal how mechanical forces orchestrate coordinated epigenetic responses that ultimately determine cell behavior and fate decisions. Future studies should leverage these integrative multi-omic strategies to systematically dissect how distinct mechanical cues are encoded in the epigenome and translated into functional cellular outcomes.

## Conclusion and future directions

The remarkable progress in understanding nuclear mechanotransduction has been driven by technological innovations spanning multiple disciplines from biophysics and materials science to genomics and computational biology. The integration of these complementary approaches has transformed our view of the nucleus from a passive recipient of biochemical signals to an active mechanosensory organelle that dynamically responds to physical forces through coordinated genomic, epigenomic, and transcriptomic changes. As these technologies continue to advance and converge, we anticipate even deeper insights into the molecular mechanisms connecting extracellular mechanical cues to nuclear organization and epigenetic regulation.

Despite growing interest, the precise roles of individual nuclear membrane constituents in transducing forces from the cytoplasm into the nucleus remain largely unclear. Deciphering the force dynamics across nuclear envelope components is important to understand this process, where a wide range of FRET (Fluorescence Resonance Energy Transfer)-based tension sensors have been constructed targeting nuclear envelope proteins [308–312]. However, these tools face key limitations, including a restricted range of measurable forces determined by the selected FRET pairs, sensor stability, and the complexity of imaging system optimization, which leave ample room for future development.

Moreover, the integration of machine learning (ML) algorithms in mechanobiology is emerging as a promising approach for predicting the mechanophenotype of cells [313,314]. Applying ML to interpret nuclear mechanotransduction holds great promise for uncovering hidden patterns and mechanisms. Nonetheless, the success of such models relies on the availability of large, high-quality experimental datasets and their accuracy may be hindered by the intrinsic variability of cell mechanics, warranting future investigation. In the field of sequencing technologies, scHi-C is still limited by intricate protocols, high computational demand, and complex downstream bioinformatic analysis.

Particularly promising are emerging capabilities for simultaneous measurement of mechanical forces, real-time visualization of nuclear responses, and comprehensive mapping of resulting epigenetic changes to create truly integrated views of mechanotransduction from initial force application to ultimate gene regulatory outcomes. These technological advances not only enhance our fundamental understanding of mechanobiology, but also open new avenues for controlling cell fate through mechanical cues, with profound implications for tissue engineering, regenerative medicine, and our understanding of mechanically influenced diseases.

## Data Availability

Data sharing is not applicable to this article as no new data were created or analyzed in this study.
